# Supplementary material for: Deformation-Thermal Co-Induced Ferromagnetism of Austenite Nanocrystalline FeCoCr Powders for Strong Microwave Absorption
Source: Nanomaterials (Basel). 2022 Jun 30;12(13):2263. doi: 10.3390/nano12132263 (PMC9268570; doi:10.3390/nano12132263)
Supplement: Supplementary file 1 [file nanomaterials-12-02263-s001.zip › nanomaterials-1739023-supplementary.pdf]

nanomaterials

Supplementary Material for:

Deformation-Thermal Co-Induced Ferromagnetism of Austenite Nanocrystalline FeCoCr Powders for Strong Microwave Absorption

Ziwen Fu [1], Zhihong Chen [1,2,*], Rui Wang [2], Hanyan Xiao [2], Jun Wang [2], Hao Yang [1], Yueting Shi [1], Wei Li [2] and Jianguo Guan [2,*]

[1] School of Science, Wuhan University of Technology, Wuhan 430070, China; ziwenfu@whut.edu.cn (Z.F.); 261222@whut.edu.cn (H.Y.); 261179@whut.edu.cn (Y.S.)
[2] State Key Laboratory of Advanced Technology for Materials Synthesis and Processing, Wuhan University of Technology, Wuhan 430070, China; 303565@whut.edu.cn (R.W.); hanyan_xiao@whut.edu.cn (H.X.); 303841@whut.edu.cn (J.W.); wellee@whut.edu.cn (W.L.)

* Correspondence: z_chen@whut.edu.cn (Z.C.); guanjg@whut.edu.cn (J.G.)

Table S1. Element composition of FeCoCr powders.

Sample	Fe (wt. %)	Co (wt. %)	Cr (wt. %)
Raw powder	42.88	41.85	15.27
Milling 4 h	42.37	42.28	15.35

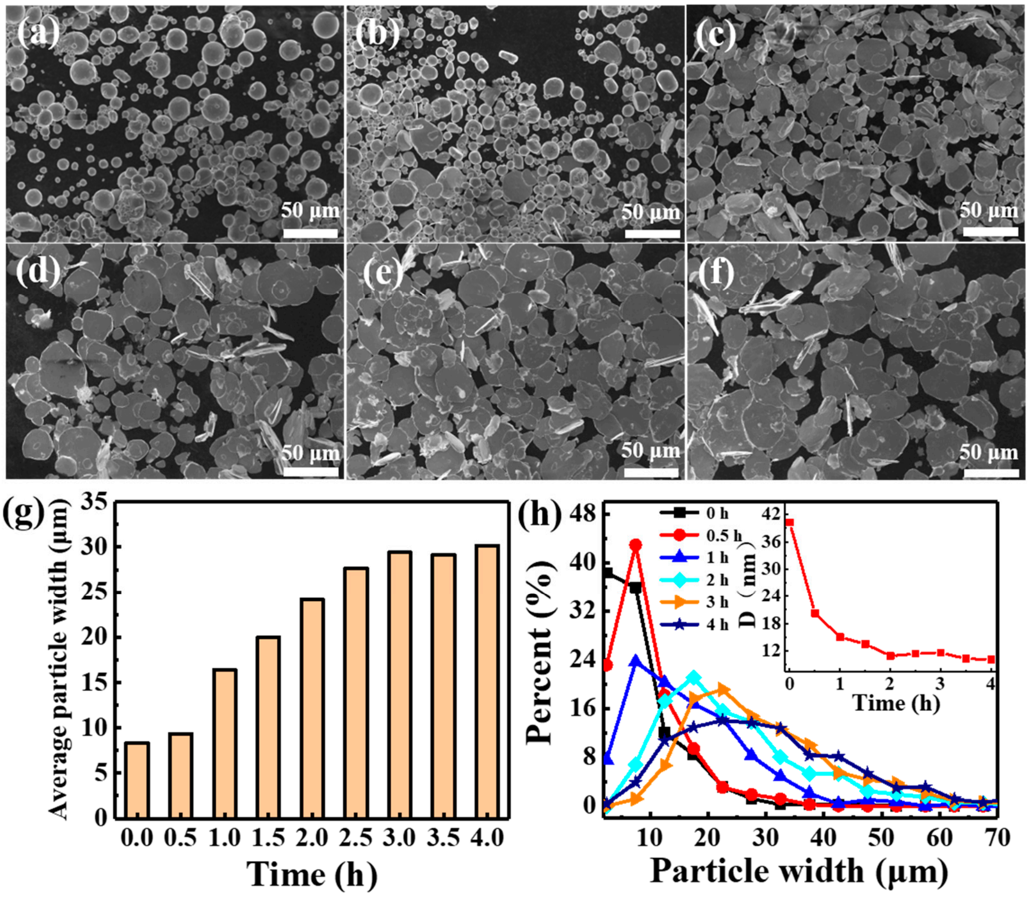

Figure S1. (**a-f**) SEM images of FeCoCr particles after ball milling for 0, 0.5, 1, 2, 3 and 4 h, respectively; (**g**) Change of particle width at different milling time; (**h**) Change of particle width distribution at different milling time. Inset in (h) is the change of grain size at different milling time.

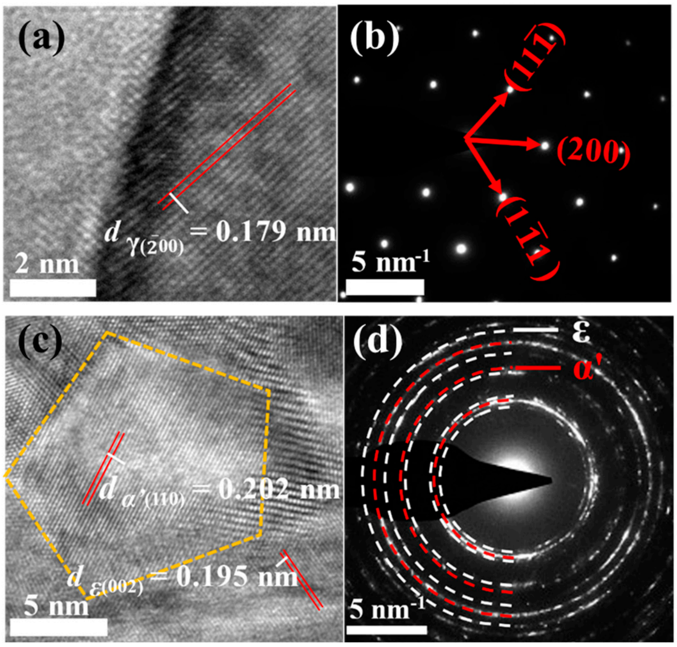

Figure S2. (**a**) High-resolution TEM image and (**b**) SEAD pattern of raw FeCoCr powder; (**c**) High-resolution TEM image and (**d**) SAED pattern of FeCoCr powder milled for 4 h.

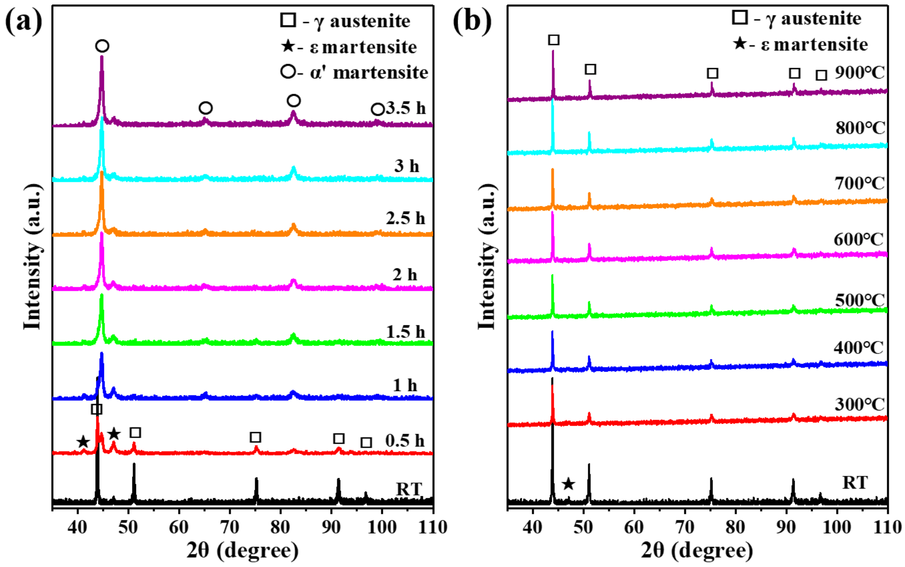

Figure S3. XRD spectra of FeCoCr powders (**a**) after ball milling for different time and (**b**) after heat treatment at different time without deformation.

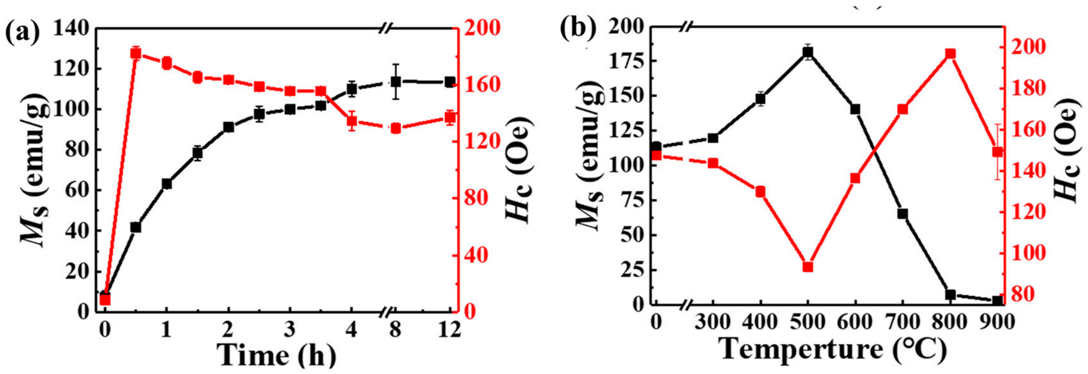

Figure S4. Evolution of saturation magnetization (M_s) and coercivity (H_c) during (**a**) ball milling and (**b**) subsequent heat treatment.

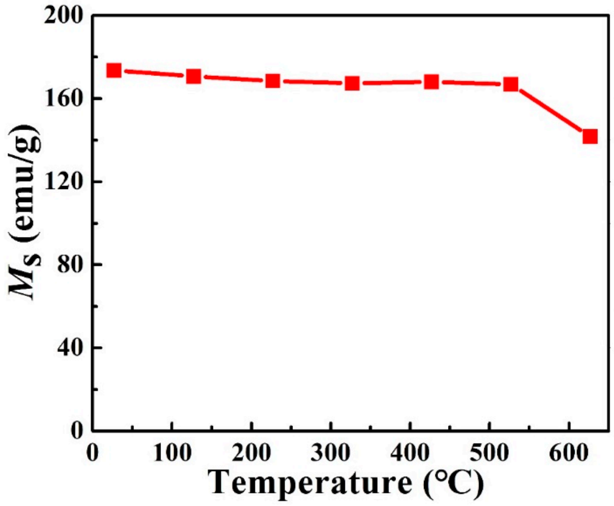

Figure S5. Change of M_s with the increase of temperature for FeCoCr powders after milling for 4 h and heat treatment at 500 °C.

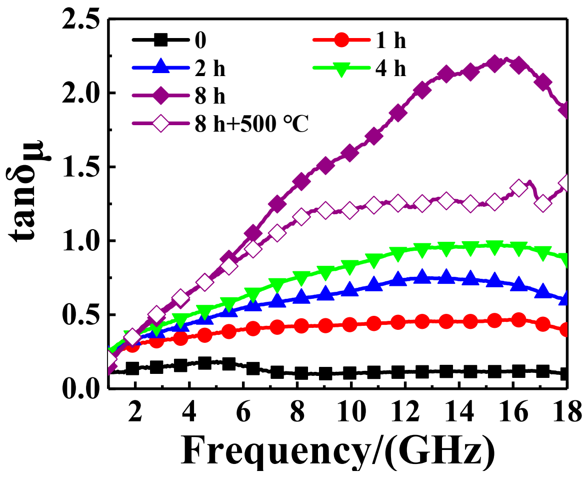

Figure S6. Change of magnetic loss tangents ($\tan\delta_\mu$) in FeCoCr powders at different milling time.
